# Clinical Retrospect

**Published:** 1799-07

**Authors:** 


*As a Supplement to the "CRITICAL RETROSPECT" we have regularly given in the preceding Numbers, we are at present, for want of room, obliged to defer the account of ſeveral intereſting works to the next volume, and to announce only the following :*

### BELLA, CRUENTA BELLA ! !

I. *Remarks on Mr. John Bell's Anatomy of the Heart and Arteries.* By Jonathan Dawplucker, Eſq. ? *London.* Robinſons. Price 2*s.* 6*d.* 1799.

II. *Number Second, being Remarks on the Firſt Volume of* Mr. Benjamin Bell's *Syſtem of Surgery :* By Jonathan Dawplucker, Eſq. ?? *London.* Printed in the year M.DCC.XCIX.

It is not without a degree of reluctance, mingled with painful reflections on the conſequences likely to reſult from the acrimonious diſpute ſubſiſting between the meritorious writers, who are the ſubjects of the two pamphlets now before the public, that we undertake to notice theſe polemical productions.

No. I. is written in a ſtrain of ſatirical humour, which, although pointed, and often witty, does not in general overſtep the bounds of modeſty and decorum.

The author endeavours to refute a great number of Mr. John Bell's aſſertions and opinions, for which we refer the reader to the pamphlet itſelf.

No. II. profeſſes to be a continuation of a work begun in the preceding pamphlet, and to be the production of the ſame author ; but it will be obvious to every reader, that they are the works of very different authors ; and that the *ſecond* is an attempt to take vengeance on the author of the firſt.—It is written with every mark of hoſtile virulence, and well calculated to produce irreconcileable enmity between the two authors and their friends. How far the provocation can juſtify ſuch an attempt, we leave to the deciſion of an impartial public ; and would gladly draw a veil over all ſuch controverſies.

---

### CÆSAREAN OPERATION.

*A Defence of the Cæſarean Operation, with Obſervations on Embryulcia, and the Section of Symphyſis Pubis, addreſſed to Mr. W. Simmons, of Mancheſter, Author of Reflections on the propriety of performing the Cæſarean Operation : Containing ſome new Caſes, and illuſtrated by ſeven Engravings.* By JOHN HULL, *M. D.* &c. &c. *Mancheſter,* Clarke ; *London,* Bickerſtaff ; *Edinburgh,* Mudie.

This Volume is intended as a refutation of the principles advanced in the treatiſe, quoted in the title-page, on the merits of which we have already pronounced our opinion in the third Number of this Journal, pp. 308 and 309.—We did not indeed, then, think proper to take notice of the unfortunate diſpute ſubſiſting between the two authors, any further than what related to the point in queſtion. Our limited room will not permit us to enter into a detailed account of this conteſted ſubject ; but, in juſtice to the author of the volume before us, we allow him the merit of having furniſhed a very learned treatiſe, abounding with hiſtorical and critical quotations. Whether he has exhauſted the ſubject, or even elucidated the queſtion, ſo as to enable us to form a concluſive opinion on this intricate operation, we ſhall not venture to decide.

In

In conſequence of this work, which appeared after the publication of Mr. Simmons's "*Reflections*," whom therefore Dr. Hull conſiders as the aggreſſor, we have had occaſion to notice another pamphlet written by Mr. S. in anſwer to the Doctor's '*Defence*,' of which we have already given a ſhort account in our laſt number, p. 410 and 411. In reply to this pamphlet, the ſpirit and temper of which we cannot admire, Dr. Hull has been induced to publiſh—

*Obſervations on Mr. Simmons's Detection, &c. &c. with a Defence of the Cæſarean Operation derived from Authorities, &c. &c. A Deſcription of the Female Pelvis ; an examination of Dr. Osborne's opinions relative to Embryulcia ; and an account of the method of delivery by Embryotomy. Illuſtrated by numerous Engravings : by* JOHN HULL, M. D. *Mancheſter, &c.*

It will not be expected by *temperate* readers, that we ſhould add another ſentence tending to extend the boundaries of this literary warfare, as we have no inclination to change our opinion quoted in the account of the preceding article; hoping ſincerely that this production will terminate the diſpute, without carrying it to any other but a *literary forum.*

